# The association between alcohol, betel nut, and cigarette use with hepatitis C virus infection in Taiwan

**DOI:** 10.1038/s41598-023-50588-1

**Published:** 2023-12-27

**Authors:** Yuan-Ai Tseng, Yu-Lun Ou, Jiun-Hung Geng, Chih-Wen Wang, Da-Wei Wu, Szu-Chia Chen, Po-Liang Lu

**Affiliations:** 1 Department of Post Baccalaureate Medicine, Kaohsiung Medical University, Kaohsiung, 807 Taiwan, ROC; 2 Department of Internal Medicine, Kaohsiung Municipal Siaogang Hospital, Kaohsiung Medical University Hospital, Kaohsiung Medical University, 482, Shan-Ming Rd., Hsiao-Kang Dist., Kaohsiung, 812 Taiwan, ROC; 3 Division of Endocrinology and Metabolism, Department of Internal Medicine, Kaohsiung Medical University Hospital, Kaohsiung Medical University, Kaohsiung, Taiwan, ROC; 4 Department of Urology, Kaohsiung Municipal Siaogang Hospital, Kaohsiung Medical University Hospital, Kaohsiung Medical University, Kaohsiung, 812 Taiwan, ROC; 5 Department of Urology, Kaohsiung Medical University Hospital, Kaohsiung Medical University, Kaohsiung, 807 Taiwan, ROC; 6 Division of Hepatobiliary, Department of Internal Medicine, Kaohsiung Medical University Hospital, Kaohsiung Medical University, Kaohsiung, Taiwan, ROC; 7 Division of Pulmonary and Critical Care Medicine, Department of Internal Medicine, Kaohsiung Medical University Hospital, Kaohsiung Medical University, Kaohsiung, 807 Taiwan, ROC; 8 Division of Nephrology, Department of Internal Medicine, Kaohsiung Medical University Hospital, Kaohsiung Medical University, Kaohsiung, 807 Taiwan, ROC; 9 Division of Infectious Diseases, Department of Internal Medicine, Kaohsiung Medical University Hospital, Kaohsiung 807, No.100, Tzyou 1st Rd., Sanmin Dist., Kaohsiung City, 80756 Taiwan, ROC; 10 Faculty of Medicine, College of Medicine, Kaohsiung Medical University, Kaohsiung, 807 Taiwan, ROC; 11 Center for Liquid Biopsy and Cohort Research, Kaohsiung Medical University, Kaohsiung, Taiwan, ROC

**Keywords:** Health care, Medical research, Risk factors

## Abstract

Hepatitis C virus (HCV) infection may cause chronic liver disease, liver cirrhosis, and liver cancer. It has been reported to associate with habits including alcohol, betel nut and cigarette use. We aimed to investigate the association between alcohol, betel nut, and cigarette use with HCV infection in Taiwan and to explore their effects. A total of 121,421 participants were enrolled from the Taiwan Biobank. They were stratified into two groups according to whether they had (*n* = 2750; 2.3%) or did not have (*n* = 118,671; 97.7%) HCV infection. All participants were also classified into four groups according to the number of habits, including a history of alcohol drinking, betel nut chewing, and cigarette smoking. There were 85,406 (no habit), 24,299 (one habit), 8659 (two habits), and 3057 (three habits) participants in the four groups, respectively. Multivariable analysis showed that the participants who had an alcohol drinking history (odds ratio [OR] 1.568; 95% confidence interval [CI] 1.388–1.773; *p* < 0.001), betel nut chewing history (OR 1.664; 95% CI 1.445–1.917; *p* < 0.001), cigarette smoking history (OR 1.387; 95% CI 1.254–1.535; *p* < 0.001), were significantly associated with HCV infection. Furthermore, the participants were classified into four groups according to the number of habits as follows: 85,406 (no habit), 24,299 (one habit), 8659 (two habits), and 3057 (three habits). The HCV infection rates in these four groups were 2.11%, 2.14%, 3.23%, and 4.78%, respectively. Compared to the participants with no or one habit, those with two habits had a higher HCV infection rate (all *p* < 0.001). In addition, compared to the participants who had no, one or two habits, those who had three habits also had higher HCV infection rates (all *p* < 0.001). The participants who had three habits had the highest prevalence of HCV infection. In an era when most HCV can be cured, understanding the epidemiology link between habits and HCV may help the case finding.

## Introduction

Taiwan has a high prevalence of hepatitis C virus (HCV) infection, with an estimated prevalence of 2.09% (95% CI, 1.60–2.77%) in the general population, which is higher than the global prevalence^1^. In 2020, the incidence of HCV infection was around 2.55 per 100,000 population in Taiwan^2^. The main risk factors for the transmission of HCV are intravenous drug use, blood transfusion, and sexual behavior^3–6^. HCV infection is a significant cause of hepatic fibrosis, cirrhosis, and hepatocellular carcinoma (HCC)^7–9^. In recent years, antiviral therapies for HCV infection have led to very high cure rates and few side effects^10^. Direct-acting antiviral agents for hepatitis C-related liver cirrhosis have proved to be an excellent treatment, with high tolerability and relatively low rates of serious adverse events^11^. In addition, attaining a treatment-related sustained virologic response among persons with HCV is associated with a reduction in the relative risk for HCC^12^. Therefore, identifying risk factors for HCV as early as possible is crucial to identify cases and for subsequent treatment.

Cigarette smoking, alcohol consumption, and betel nut chewing are common habits in Taiwan^13–15^. These behaviors may contribute to the development of oral cancer, esophageal cancer, stomach cancer, and liver cancer^16–19^. Moreover, a synergistic risk effect of these behaviors on oral cancer, esophageal cancer and HCC has been reported^16,19,20^. HCV infection is linked to blood exposure and injecting drug users, which were more common among cigarette smokers. Furthermore, a high proportion of cigarette smokers were habitual drinkers and betel nut chewers^14,21,22^. The use of these different substances was found to cluster. Previous studies have also reported that alcohol drinking, betel nut chewing, and cigarette smoking were independently associated with HCV infection^23–27^. However, the association between these three habits and HCV infection remains poorly defined.

Therefore, the aim of this study was to investigate the associations between alcohol drinking, betel nut chewing and cigarette smoking with HCV infection in around 120,000 participants in the Taiwan Biobank (TWB). We further investigated the effect between alcohol, betel nut and cigarette use on HCV infection.

## Materials and methods

### TWB

To enhance biomedical and epidemiological research in Taiwan, the TWB was launched by the government in 2012 as an ongoing prospective study of men and women aged 30–70 years recruited from approximately 30 centers around the country. Comprehensive genomic and phenotypic data are collected and recorded for each participant at enrollment and during follow-up visits through structured questionnaires, physical examinations, and urine and blood tests^28,29^. We used first enrolled data of TWB for further analysis. All participants in the TWB provided written informed consent before enrollment.

### Demographic, lifestyle, laboratory and medical data

Information obtained through the TWB structured questionnaires included sex and age, and habits for alcohol drinking, betel nut chewing, and cigarette smoking. None of the enrollees in the TWB have a history of cancer. A history of other diseases such as diabetes mellitus (DM) and hypertension was recorded. Weight and height were measured during the physical examinations, and the body mass index (BMI; kg/m^2^) was recorded. In addition, analysis of the urine and blood samples provided data on: hemoglobin, fasting glucose, triglycerides, total cholesterol, uric acid, aspartate aminotransferase (AST), alanine aminotransferase (ALT) and estimated glomerular filtration rate (eGFR), calculated using the Modification of Diet in Renal Disease equation^30^. Chemiluminescence was used to test for anti-HCV antibodies (ADVIA Centaur, Siemens).

### Assessment of alcohol drinking, betel nut chewing, and cigarette smoking history

The definitions of habits for alcohol drinking, betel nut chewing, and cigarette smoking history were as follows. For betel nut, those who had chewed ≥ 1 betel nut/per week for at least 1 year were defined as ever chewers, and those who had smoked ≥ 1 cigarette per day for at least 1 year were defined as ever smokers. For alcohol drinking, those who had consumed any alcoholic beverage > 4 times a week for at least 1 year were defined as ever drinkers.

The frequency of betel nut chewing in the ever chewers was recorded as follows: 1–3 days/month (1 point); 1–2 days/week (2 points); 3–5 days/week (3 points); 6–7 days (4 points). The daily amount of betel nut chewing was then recorded as follows: < 10 quids (1 point); 10–20 quids (2 points); 21–30 quids (3 points); ≥ 31 quids (4 points). Finally, the cumulative dose was calculated as: years of chewing betel nut × frequency score × daily amount score.

### Study participants

**Figure 1:**
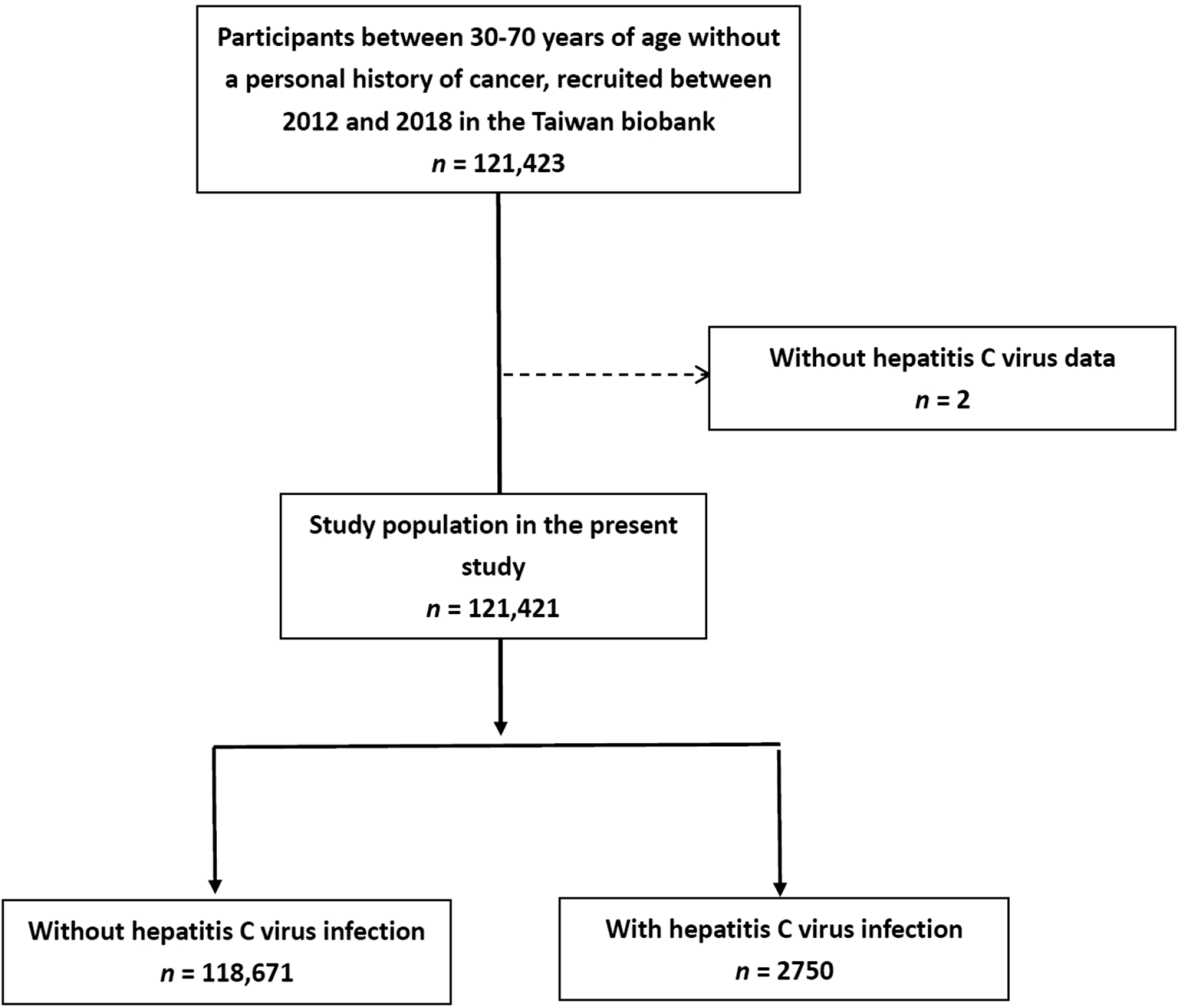
Flowchart of study population.

Of 121,423 individuals in the TWB, 2 did not have data on HCV and were excluded. The remaining 121,421 individuals (males: 43,636; females: 77,785; mean age: 49.9 ± 11.0 years) were enrolled, and divided into two groups according to whether they had (*n* = 2750; 2.3%) or did not have (*n* = 118,671; 97.7%) HCV infection (Fig. 1).

### Ethics statement

The study was conducted according to the Declaration of Helsinki, and it was granted approval by the Institutional Review Board of Kaohsiung Medical University Hospital (KMUHIRB-E(I)-20,210,058), and the TWB was granted approval by the IRB on Biomedical Science Research, Academia Sinica, Taiwan and the Ethics and Governance Council of the TWB.

### Statistical analysis

Data are presented as n (%) or as mean ± SD. Continuous variables were compared using the independent t test, and categorical variables were compared using the chi-square test. Associations between alcohol drinking, betel nut chewing, cigarette smoking, and their characteristics with HCV infection were evaluated using multivariable logistic regression analysis. To avoid bias of demographic and characteristic differences between HCV (−) and HCV (+) groups, we further performed 1:10 matching using propensity score matching. In order to exclude the possibility that habit was associated with different sex and age group, we further performed subgroup analysis (male and female participants, and participants aged ≥ 50 and < 50 years). Besides, subgroup analysis of the associations between the number of habits and HCV infection were evaluated using multivariable logistic regression analysis. A *p* value of < 0.05 was considered to indicate a statistically significant difference. Statistical analysis was performed using SPSS version 26.0 for Windows (SPSS Inc., Armonk, NY, USA).

### Ethics approval and consent to participate

The study was conducted according to the Declaration of Helsinki, and it was granted approval by the Institutional Review Board of Kaohsiung Medical University Hospital (KMUHIRB-E(I)-20,210,058), and the TWB was granted approval by the IRB on Biomedical Science Research, Academia Sinica, Taiwan and the Ethics and Governance Council of the TWB. All participants in the TWB provide written informed consent before enrollment.

## Results

### Comparisons of clinical characteristics between the with and without HCV groups

The group with HCV infection group were older and had more males than the without HCV infection group. In addition, those with HCV infection had higher rates of DM, hypertension, alcohol drinking, betel nut chewing, and cigarette smoking, lower rates of education higher than senior high schools, and higher levels of fasting glucose, hemoglobin, AST, ALT, and uric acid, and lower total cholesterol and eGFR than those without HCV infection (Table 1).

**Table 1:** Comparison of clinical characteristics among participants with and without hepatitis C virus infection (*n* = 121,421).

Characteristics	HCV (−) (*n* = 118,671)	HCV (+) (*n* = 2750)	*p*
Age (year)	49.8 ± 11.0	54.3 ± 9.7	< 0.001
Male (%)	35.9	36.3	< 0.001
DM (%)	5.1	7.9	< 0.001
Hypertension (%)	12.1	17.0	< 0.001
Alcohol drinking history (%)	8.4	13.2	< 0.001
Betel nut chewing history (%)	6.0	10.4	< 0.001
Cigarette smoking history (%)	27.2	31.1	< 0.001
Education higher than senior high schools (%)	87.7	71.6	< 0.001
Body mass index (kg/m^2^)	24.2 ± 3.8	24.3 ± 3.7	0.095
Laboratory parameters			
Fasting glucose (mg/dL)	95.9 ± 20.6	98.6 ± 24.8	< 0.001
Hemoglobin (g/dL)	13.8 ± 1.6	13.9 ± 1.6	< 0.001
Triglyceride (mg/dL)	115.7 ± 93.8	113.6 ± 102.2	0.288
Total cholesterol (mg/dL)	195.8 ± 35.8	187.8 ± 37.0	< 0.001
AST (U/L)	24.8 ± 12.1	32.9 ± 25.5	< 0.001
ALT (U/L)	23.6 ± 20.2	34.0 ± 40.3	< 0.001
eGFR (mL/min/1.73 m^2^)	103.3 ± 23.9	100.4 ± 24.1	< 0.001
Uric acid (mg/dL)	5.4 ± 1.4	5.5 ± 1.4	< 0.001

*HCV* hepatitis C virus, *DM* diabetes mellitus, *AST* spartate aminotransferase, *ALT* alanine aminotransferase, *eGFR* estimated glomerular filtration rate.

### Determinants of HCV infection

The results of multivariable logistic regression analysis for the factors associated with HCV infection in the whole cohort (*n* = 121,421) are shown in Table 2. We performed five models to show the factors associated with HCV infection. Model 1: Adjusted alcohol drinking history and covariates of age, sex, diabetes, hypertension, fasting glucose, hemoglobin, total cholesterol, AST, ALT, eGFR and uric acid (significant variables in Table 1). Model 2: Adjusted betel nut chewing history and covariates. Model 3: Adjusted cigarette smoking history and covariates. Model 4: Adjusted alcohol drinking, betel nut chewing, and cigarette smoking history and covariates. Model 5: Interaction test was conducted by including multiplicative interaction terms in the multiple logistic regression model. After adjusting for age, sex, DM, hypertension, alcohol drinking, betel nut chewing, and cigarette smoking, education status, fasting glucose, hemoglobin, total cholesterol, AST, ALT, eGFR and uric acid (significant variables in Table 1), alcohol drinking history (odds ratio [OR] 1.568; 95% confidence interval [CI] 1.388–1.773; *p* < 0.001) in model 1, betel nut chewing history (OR 1.664; 95% CI 1.445–1.917; *p* < 0.001) in model 2, cigarette smoking history (OR 1.387; 95% CI 1.254–1.535; *p* < 0.001) in model 3 were significantly associated with HCV infection.

**Table 2:** Multivariable logistic regression analysis of factors associated with hepatitis C virus infection (*n* = 121,421).

Variables	Model 1	*p*	Model 2	*p*	Model 3	*p*	Model 4	*p*	Model 5
	Adjusted OR (95% CI)		Adjusted OR (95% CI)		Adjusted OR (95% CI)		Adjusted OR (95% CI)		*p*
Intercept									< 0.001
Age (per 1 year)	1.034 (1.029–1.038)	< 0.001	1.034 (1.029–1.038)	< 0.001	1.034 (1.030–1.039)	< 0.001	1.035 (1.030–1.039)	< 0.001	< 0.001
Male (*vs.* female)	0.702 (0.627–0.787)	< 0.001	0.686 (0.601–0.770)	< 0.001	0.646 (0.571–0.731)	< 0.001	0.601 (0.530–0.682)	< 0.001	< 0.001
DM	0.883 (0.748–1.042)	0.141	0.878 (0.744–1.036)	0.123	0.878 (0.744–1.036)	0.123	0.875 (0.741–1.033)	0.1152-	0.113
Hypertension	0.915 (0.820–1.020)	0.110	0.918 (0.824–1.024)	0.126	0.921 (0.826–1.027)	0.140	0.909 (0.815–1.014)	0.0871-	0.087
Alcohol drinking history	1.568 (1.388–1.773)	< 0.001					1.364 (1.197–1.554)	< 0.001	0.114
Betel nut chewing history			1.664 (1.445–1.917)	< 0.001			1.387 (1.192–1.613)	< 0.001	0.317
Cigarette smoking history					1.387 (1.254–1.535)	< 0.001	1.233 (1.106–1.375)	< 0.001	0.001
Education Higher than senior high schools	0.480 (0.438–0.527)	< 0.001	0.491 (0.448–0.539)	< 0.001	0.476 (0.434–0.522)	< 0.001	0.494 (0.450–0.542)	< 0.001	< 0.001
Fasting glucose (per 1 SD mg/dL)	1.017 (0.978–1.057)	0.407	1.016 (0.977–1.057)	0.418	1.017 (0.978–1.058)	0.393	1.014 (0.975–1.055)	0.481	0.476
Hemoglobin (per 1 SD g/dL)	1.150 (1.092–1.212)	< 0.001	1.151 (1.092–1.212)	< 0.001	1.151 (1.092–1.213)	< 0.001	1.148 (1.090–1.210)	< 0.001	< 0.001
Total cholesterol (per 1 SD mg/dL)	0.707 (0.677–0.738)	< 0.001	0.707 (0.678–0.738)	< 0.001	0.707 (0.677–0.738)	< 0.001	0.707 (0.677–0.738)	< 0.001	< 0.001
AST (per 1 SD U/L)	1.161 (1.113–1.210)	< 0.001	1.167 (1.119–1.217)	< 0.001	1.171 (1.123–1.221)	< 0.001	1.158 (1.111–1.207)	< 0.001	< 0.001
ALT (per 1 SD U/L)	1.065 (1.017–1.116)	0.007	1.058 (1.010–1.108)	0.018	1.057 (1.008–1.107)	0.0210	1.067 (1.019–1.117)	0.006	0.006
eGFR (per 1 SD mL/min/1.73 m^2^)	0.973 (0.931–1.017)	0.222	0.976 (0.934–1.021)	0.290	0.978 (0.935–1.022)	0.313	0.973 (0.931–1.017)	0.223	0.221
Uric acid (per 1 SD mg/dL)	1.020 (0.973–1.069)	0.410	1.026 (0.979–1.075)	0.278	1.027 (0.980–1.076)	0.268	1.018 (0.971–1.067)	0.454	0.459
Interaction term									
Alcohol and Betel nut									0.801
Alcohol and Cigarette									0.397
Betel nut and Cigarette									0.979
Alcohol, Betel nut, and Cigarette									0.772

Values expressed as odds ratio (OR) and 95% confidence interval (CI). *HCV* hepatitis C virus, *DM* diabetes mellitus, *SD* standard deviation, *AST* aspartate aminotransferase, *ALT* alanine aminotransferase, *eGFR* estimated glomerular filtration rate.

Adjusted for age, sex, diabetes, hypertension, alcohol drinking, betel nut chewing, and cigarette smoking history, fasting glucose, hemoglobin, total cholesterol, AST, ALT, eGFR and uric acid (significant variables in Table 1).

Model 1: Adjusted alcohol drinking history and covariates of age, sex, diabetes, hypertension, fasting glucose, hemoglobin, total cholesterol, AST, ALT, eGFR and uric acid (significant variables in Table 1).

Model 2: Adjusted betel nut chewing history and covariates.

Model 3: Adjusted cigarette smoking history and covariates.

Model 4: Adjusted alcohol drinking, betel nut chewing, and cigarette smoking history and covariates.

Model 5: Interaction test was conducted by including multiplicative interaction terms in the multiple logistic regression model.

We then performed a sub-analysis of the determinants of HCV infection in the participants who chewed betel nut (n = 7355). Multivariable logistic regression analysis showed that a high cumulative dose (per 1 year × frequency × daily score; OR = 1.001; 95% CI 1.000–1.003; *p* = 0.042) was significantly associated with HCV infection.

### Association of subgroup of habits combination associated with HCV infection

The results of multivariable logistic regression analysis for subgroup of habits combination associated with HCV infection in the whole cohort (n = 121,421) are shown in Table 3. After multiple adjustment, compared to the group of alcohol (−) betel nut (−) cigarette (−), the group of alcohol (−) betel nut (−) cigarette (+) (OR 1.212; 95% CI 1.077–1.365; *p* = 0.001), the group of alcohol (+) betel nut (+) cigarette (−) (OR 2.337; 95% CI 1.019–5.360; *p* = 0.045), the group of alcohol (+) betel nut (−) cigarette (+) (OR 1.770; 95% CI 1.473–2.127; *p* < 0.001), the group of alcohol (−) betel nut (+) cigarette (+) (OR 1.898; 95% CI 1.546–2.331; *p* < 0.001), and the group of alcohol (+) betel nut (+) cigarette (+) (OR 2.677; 95% CI 2.201–3.256; *p* < 0.001) were significantly associated with HCV infection.

**Table 3:** Multivariable logistic regression analysis of subgroup of habits combination associated with hepatitis C virus infection (*n* = 121,421).

Variables	Multivariable (HCV)	
	Odds ratio (95% CI)	*p*
Alcohol (−) Betel nut (−) Cigarette (−)	Reference	
Alcohol (+) Betel nut (−) Cigarette (−)	1.237 (0.950–1.611)	0.115
Alcohol (−) Betel nut (+) Cigarette (−)	1.397 (0.725–2.695)	0.318
Alcohol (−) Betel nut (−) Cigarette (+)	1.216 (1.080–1.369)	0.001
Alcohol (+) Betel nut (+) Cigarette (−)	1.988 (0.864–4.578)	0.106
Alcohol (+) Betel nut (−) Cigarette (+)	1.731 (1.441–2.081)	< 0.001
Alcohol (−) Betel nut (+) Cigarette (+)	1.685 (1.371–2.071)	< 0.001
Alcohol (+) Betel nut (+) Cigarette (+)	2.335 (1.917–2.844)	< 0.001

Values expressed as odds ratio and 95% confidence interval (CI).

*HCV* hepatitis C virus.

Adjusted for age, sex, diabetes, hypertension, education status, fasting glucose, hemoglobin, total cholesterol, AST, ALT, eGFR and uric acid (significant variables in Table 1).

We have further performed the synergy index. The synergy index was calculated as (odds ratio for Alcohol and Betel nut − 1) ÷ ([odds ratio for Alcohol or Betel nut] − 2). Synergy index (Alcohol and Betel nut) = $\frac{1.988 - 1}{{\left( {1.237 + 1.397} \right) - 2}} =$ 1.56. The synergy index was calculated as (odds ratio for Alcohol and Cigarette − 1) ÷ ([odds ratio for Alcohol or Cigarette] − 2). Synergy index (Alcohol and Cigarette) = $\frac{1.731 - 1}{{\left( {1.237 + 1.216} \right) - 2}} =$ 1.61. The synergy index was calculated as (odds ratio for Betel nut and Cigarette − 1) ÷ ([odds ratio for Betel nut or Cigarette] − 2). Synergy index (Betel nut and Cigarette) = $\frac{1.685 - 1}{{\left( {1.397 + 1.216} \right) - 2}} =$ 1.12. The values of synergy index are all greater than 1. Our results revealed that alcohol drinking, betel nut chewing, and cigarette smoking history had an additive interaction associated with hepatitis C virus infection.

To avoid bias of demographic and characteristic differences between HCV (−) and HCV (+) groups, we have added 1:10 matching using propensity score matching (Table S1). Compared to the group of alcohol (−) betel nut (−) cigarette (−), the group of alcohol (+) betel nut (−) cigarette (+) (OR 1.426; 95% CI 1.191–1.707; *p* < 0.001), the group of alcohol (−) betel nut (+) cigarette (+) (OR 1.299; 95% CI 1.066–1.582; *p* = 0.009), and the group of alcohol (+) betel nut (+) cigarette (+) (OR 1.697; 95% CI 1.405–2.050; *p* < 0.001) were significantly associated with HCV infection (Table S2).

### Association of the number of habits with HCV infection

**Figure 2:**
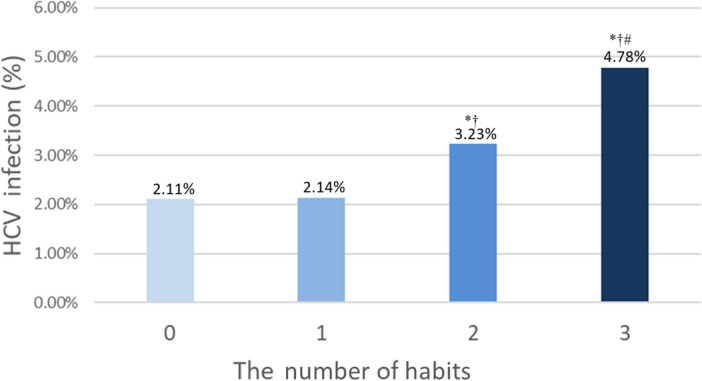
The hepatitis C virus infection rate among 4 study groups according to the number of habits have. ^*^p < 0.05 compared no habit; ^†^*p* < 0.05 compared with one habit; ^#^*p* < 0.05 compared with two habits.

The participants were classified into four groups according to the number of habits as follows: 85,406 (no habit), 24,299 (one habit), 8659 (two habits), and 3057 (three habits). The HCV infection rates in these four groups were 2.11%, 2.14%, 3.23%, and 4.78%, respectively (Fig. 2). Compared to the participants who had no or one habit, those who had two habits had higher HCV infection rate (all *p* < 0.001). In addition, compared to the participants who had no, one or two habits, those who had three habits also had higher HCV infection rates (all *p* < 0.001). The participants who had three habits had the highest prevalence of HCV infection.

### Subgroup analysis of the associations between the number of habits and HCV infection of different sex

Table 4 shows the subgroup analysis of associations between the number of habits and HCV infection using multivariable logistic regression analysis. The male participants (*n* = 42,636) who had two habits (vs. no habit; OR 1.599; 95% CI 1.338–1.911; *p* < 0.001), and three habits (vs. no habit; OR 2.313; 95% CI 1.870–2.862; *p* < 0.001) were significantly associated with HCV infection. However, the male participants had one habit was not (*p* = 0.068). The female participants (*n* = 77,785) who had one habit (vs. no habit; OR 1.312; 95% CI 1.120–1.535; *p* < 0.001), two habits (vs. no habit; OR 2.334; 95% CI 1.721–3.167; *p* < 0.001), and three habits (vs. no habit; OR 3.371; 95% CI 1.189–9.557; *p* < 0.001) were significantly associated with HCV infection.

**Table 4:** Subgroup analysis of association of the number of habits and hepatitis C virus infection in multivariable logistic regression analysis.

Habit	Multivariable (Male, *n* = 43,636)		Multivariable (Female, *n* = 77,785)	
	Odds ratio (95% CI)	Odds ratio (95% CI)	Odds ratio (95% CI)	*p*
No habit	Reference	Reference	Reference	
One habit	1.161 (0.989–1.364)	0.068	1.312 (1.120–1.535)	< 0.001
Two habits	1.599 (1.338–1.911)	< 0.001	2.334 (1.721–3.167)	< 0.001
Three habits	2.313 (1.870–2.862)	< 0.001	3.371 (1.189–9.557)	< 0.001

Values expressed as odds ratio and 95% confidence interval (CI).

Abbreviations are the same as in Table 1.

Adjusted for the number of habits, age, diabetes, hypertension, education status, fasting glucose, hemoglobin, total cholesterol, AST, ALT, eGFR and uric acid.

### Subgroup analysis of the associations between the number of habits and HCV infection of different age groups

Table 5 shows the subgroup analysis of associations between the number of habits and HCV infection of different age groups using multivariable logistic regression analysis. The participants aged ≥ 50 years (*n* = 64,818), and those who had two habits (vs. no habit; OR 2.143; 95% CI 1.670–2.749; *p* < 0.001), and three habits (vs. no habit; OR 3.452; 95% CI 2.518–4.732; *p* < 0.001) were also significantly associated with HCV infection. However, the participants aged ≥ 50 years had one habit was not (*p* = 0.156). In addition, the participants aged < 50 years (*n* = 56,603), and those who had one habit (vs. no habit; OR 1.439; 95% CI 1.201–1.724; *p* < 0.001), two habits (vs. no habit; OR 1.983; 95% CI 1.544–2.548; *p* < 0.001), and three habits (vs. no habit; OR 2.979; 95% CI 2.162–4.103; *p* < 0.001) were also significantly associated with HCV infection.

**Table 5:** Subgroup analysis of association of the number of habits and hepatitis C virus infection in multivariable logistic regression analysis.

Habit	Multivariable (age ≥ 50 years old, *n* = 64,818)		Multivariable (age < 50 years old, *n* = 56,603)	
	Odds ratio (95% CI)	*p*	Odds ratio (95% CI)	*p*
No habit	Reference		Reference	
One habit	1.110 (0.961–1.283)	0.156	1.439 (1.201–1.724)	< 0.001
Two habits	1.519 (1.258–1.835)	< 0.001	1.983 (1.544–2.548)	< 0.001
Three habits	1.850 (1.435–2.386)	< 0.001	2.979 (2.162–4.103)	< 0.001

Values expressed as odds ratio and 95% confidence interval (CI). Abbreviations are the same as in Table 1.

Adjusted for the number of habits, age, sex, diabetes, hypertension, education status, fasting glucose, hemoglobin, total cholesterol, AST, ALT, eGFR and uric acid.

## Discussion

In this study, we investigated the associations between HCV infection and its characteristics among 121,421 Taiwanese participants. The results showed that alcohol drinking, betel nut chewing, and cigarette smoking were significantly associated with HCV infection. Furthermore, the more habits had, the greater the rate of HCV infection. We also found an effect of alcohol, betel nut, and cigarette use on HCV infection.

The first important finding of this study is that alcohol drinking was associated with HCV infection. A previous cross-sectional study reported a highly significant correlation between self-reported alcohol consumption and serum HCV ribonucleic acid levels (*r* = 0.26, *p* < 0.0001), suggesting that greater alcohol consumption was associated with higher virus levels in the blood^31^. There are several potential mechanisms for this association. First, alcohol may impair cellular immunity and inhibit the efficacy of antiviral therapy^32^. Thus, impaired immune function may contribute to the ability of the virus to enter and remain inside the body rather than be eliminated by immune cells. The second mechanism may be due to oxidative stress. Larrea et al. reported that HCV infection itself can lead to oxidative stress, which induces viral genome heterogeneity and influences HCV propagation in the organism^33^. The mechanism of oxidative stress facilitates viral escape during treatment^34^, and escape from the immune system^35^. Alcohol is metabolized predominantly in the liver and can generate free radicals that contribute to oxidative stress, which may aggravate this virus-induced oxidative stress^36^. Therefore, impaired immune function and oxidative stress may play important roles in the association between alcohol drinking and HCV infection. Furthermore, Charles et al. found that people with alcohol abuse disorder had higher rates of HCV infection than controls, even in those with no other classical risk factors for HCV infection such as intravenous drug abuse or blood transfusions^37^. Taken together, we suggest that excessive alcohol consumption may increase the risk of acquiring HCV.

The second important finding of this study is that betel nut chewing was associated with HCV infection. We further analyzed the frequency, daily amount, and cumulative dose of betel nut chewing, and found that a high cumulative dose was correlated with HCV infection. A community-based study conducted in central Taiwan was the first to show that betel nut chewing was an independent risk factor for HCV infection, with an adjusted OR of 9.12^25^. Other studies have found that the habit of betel nut chewing was an independent risk factor for HCC, and that an increased risk of HCC was associated with seropositivity for anti-HCV in Taiwan^38,39^. Taken together, these findings indirectly support that betel nut chewing is an independent risk factor for anti-HCV^25^. Further investigations are needed to elucidate the precise mechanism underlying the association between betel nut chewing and HCV infection.

Another interesting finding of this study is that cigarette smoking was associated with HCV infection. Kim et al*.* reported that the prevalence of smoking in individuals with HCV infection was nearly three times higher than in individuals without HCV infection (62.4% vs. 22.9%, *p* < 0.001) in the United States from 1999 to 2014, and that heroin use was more common in individuals with HCV infection who smoked than in individuals without HCV infection who smoked^22^. In addition, Chen et al*.* founded that the prevalence of illicit drug use ranged from 0.3% among those using no other substance to 7.1% among those using tobacco, betel quid, and alcohol among 13-to 35-year-old persons in I-Lan County, a rural area of Taiwan, supporting that tobacco smoking may have a large effect on illicit drug use^21^. Heroin users are more likely to smoke. A possible explanation is that HCV infection is linked to blood exposure, and injection drug use may increase the risk of acquiring HCV. The association between smoking and HCV may be because of the higher rate of heroin use in smokers. Another population-based study reported an association between the initiation and intensity of habits and the number of sexual partners, and suggested that the severity of alcohol and cigarette use was an indicator of a higher number of sexual partners^40^. The risk of sexually transmitted diseases and hepatitis B or C virus infection increases as the number of sexual partners increases^41^. In addition, Chuang et al*.* proposed a synergistic effect between smoking and hepatitis B or C virus infection on the risk of HCC^27^. Therefore, further investigations are needed to study the influence of cigarette smoking on HCV infection.

Lastly, we found that the more habit had, the greater the rate of HCV infection, and that there was an effect of alcohol, betel nut, and cigarette use on HCV infection. The prevalence of males and older age was significantly higher in the participants with HCV infection. To exclude the possibility that habit was associated with these groups, we further performed subgroup analysis of female participants and participants aged < 50 years, and found similar results of an effect of alcohol, betel nut and cigarette use on HCV infection. Prior studies have identified the synergistic risk effects of alcohol drinking, tobacco smoking and betel nut chewing on various cancers. Ko et al. reported that betel quid chewing, cigarette smoking and alcohol consumption were significantly related to oral cancer, which they suggested could be due to the carcinogenicity of alcoholic beverages, tobacco smoke and betel quid, with target organs including the oral cavity, pharynx, larynx and esophagus^16^. Wu et al*.* reported an interaction between these three habits on the risk of esophageal cancer in Taiwan, and suggested that betel nut plays a role in adding to the carcinogenetic effect of cigarette smoke and alcohol^20^. In addition, Wang et al. suggested that there are combined effects of habitual alcohol drinking, betel quid chewing and cigarette smoking on an increased risk of HCC^19^. Cigarette smoke contains several chemicals that are metabolized and activated as carcinogens in the liver^42^. Alcohol is a hepatotoxin which accelerates fibrosis progression and increases the risk of cirrhosis, a primary clinical predictor of HCC^43–45^. The mechanism underlying the association between betel nut chewing and HCC may also be related to safrole, which may be the reactive agent responsible for hepatocarcinogenesis^46^.

At present, alcohol drinking, betel nut chewing, and cigarette smoking history independently predicts HCV in Table 2, but it is not easy to distinguish whether independent influence is indeed on HCV. To avoid Table 2 fallacy^47^, we performed further analysis of 8 subgroups of habits combination (Table 3) to clarify that he effect of habit combinations on the association with HCV appears consistent with the results in Fig. 2 that the group of alcohol (+) betel nut (+) cigarette (+) being the highest. When any two habit factors co-exist, statistically significant effect are retained to associate with HCV. Therefore, the results revealed that the three factors do jointly affect the risk of HCV. The group of alcohol (+) betel nut (−) cigarette (−), and the group of alcohol (−) betel nut (+) cigarette (−) did not have a significant association but with a trend with HCV (Table 3). It could be caused by the number of the two groups is relatively small.

This work is the first study to show the association between alcohol, betel nut, and cigarette on the risk of HCV infection, as they were independent risk factors for HCV infection. Ko et al*.* reported that a high proportion of betel nut chewers in Taiwan were also habitual smokers and drinkers^14^. The current investigation also indicated that smoking and alcohol drinking were also independently associated with the progression of HCV infection^23,27^. In addition, we found evidence of the combined effects of chronic hepatitis virus infection and habits on chronic liver disease, and that there was a biological gradient in the risk of developing chronic liver disease, with multivariable-adjusted ORs of 4.7 and 7.9 for subjects who had 1–2 and 3 habits, respectively, compared to those who did not have the habit^48^. Betel nut chewing was recently found to be associated with HCC, esophageal cancer and gastric cancer in Taiwan^39,49,50^, and HCV infection has been considered as an important cause of advanced hepatic fibrosis and cirrhosis, with a significantly increased risk of developing HCC^51^. Thus, taken together, these findings suggest that lifestyle factors such as alcohol consumption, cigarette smoking and betel nut chewing are related to liver damage and indirectly support our findings that these three habits has effects on HCV infection. Further studies are necessary to investigate the exact mechanism by which betel nut chewing increases the risk of HCV infection in drinkers and smokers.

Our findings are strengthened by the inclusion of a large number of healthy participants enrolled from a national biobank, and controlling for confounding factors including alcohol, betel nut and cigarette use in our analyses. Nevertheless, several limitations should also be mentioned. First, as this was a cross-sectional study, it was not possible to determine the duration of HCV infection, and consequently causal relationships could not be established. Longitudinal studies are needed to clarify the association between habits and HCV infection. Second, the genotype and severity of HCV infection could not be ascertained. Third, some data associated with HCV infection was lacking, such as socioeconomic status of participants, which may influence HCV infection rate. Fourth, the alcohol history, betel nut chewing history, and smoking history were collected by questionnaire, which may result in the possibility of misclassification, and then lead to wrong analysis results. Besides, the participants of TWB may not represent the general population in Taiwan, which may result in selection bias, and then lead to underestimated or overestimated the OR. Finally, our findings may be limited by the single ethnicity of the enrolled participants. Further studies on other ethnic groups are warranted.

In conclusion, this study suggests the association between HCV infection and alcohol drinking, betel quid chewing, and cigarette smoking. Cessation programs for habits are important to prevent chronic HCV infection in Taiwan.

### Supplementary Information

Supplementary Tables.

## Data Availability

The data underlying this study are from the Taiwan Biobank Database. Due to restrictions placed on the data by the Personal Information Protection Act of Taiwan, the minimal data set cannot be made publicly available. Data may be available upon request to interested researchers. Please send data requests to: Szu-Chia Chen, PhD, MD. Division of Nephrology, Department of Internal Medicine, Kaohsiung Medical University Hospital, Kaohsiung Medical University.
