# Supplementary material for: The association between alcohol, betel nut, and cigarette use with hepatitis C virus infection in Taiwan
Source: Sci Rep. 2023 Dec 27;13:23082. doi: 10.1038/s41598-023-50588-1 (PMC10754914; doi:10.1038/s41598-023-50588-1)
Supplement: Supplementary file 1 — Supplementary Tables. [file 41598_2023_50588_MOESM1_ESM.docx]

Table S1. Comparison of clinical characteristics among participants with and without hepatitis C virus infection (1:10 matching using propensity score matching)

|  | 1:10 matching* | |  |
| --- | --- | --- | --- |
| Characteristics | HCV (-)  (*n* = 27,429) | HCV (+)  (*n* = 2,750) | *p* |
| Age (year) | 54.4 ± 10.4 | 54.3 ± 9.7 | 0.600 |
| Male (%) | 36.4 | 36.4 | 0.980 |
| DM (%) | 8.0 | 7.9 | 0.853 |
| Hypertension (%) | 16.9 | 17.0 | 0.884 |
| Education higher than senior high schools (%) | 71.8 | 71.6 | 0.812 |
| Alcohol drinking history (%) | 8.8) | 13.2 | < 0.001 |
| Betel nut chewing history (%) | 6.8) | 10.4 | < 0.001 |
| Cigarette smoking history (%) | 27.1 | 31.6 | < 0.001 |
| Body mass index (kg/m^2^) | 24.4 ± 3.7 | 24.4 ± 3.7 | 0.699 |
| Laboratory parameters |  |  |  |
| Fasting glucose (mg/dL) | 98.7 ± 24.0 | 98.6 ± 24.8 | 0.987 |
| Hemoglobin (g/dL) | 13.9 ± 1.5 | 13.9 ± 1.6 | 0.810 |
| Triglyceride (mg/dL) | 113.8 ± 75.6 | 113.6 ± 102.2 | 0.870 |
| Total cholesterol (mg/dL) | 188.6 ± 34.2 | 187.8 ± 37.0 | 0.231 |
| AST (U/L) | 25.5 ± 11.9 | 32.9 ± 25.5 | < 0.001 |
| ALT (U/L) | 23.9 ± 18.6 | 34.0 ± 40.3 | < 0.001 |
| eGFR (mL/min/1.73 m^2^) | 100.4 ± 23.7 | 100.4 ± 24.1 | 0.937 |
| Uric acid (mg/dL) | 5.5 ± 1.4 | 5.5 ± 1.4 | 0.903 |

Abbreviations. HCV, hepatitis C virus; DM, diabetes mellitus; AST, aspartate aminotransferase; ALT, alanine aminotransferase; eGFR, estimated glomerular filtration rate.

*1:10 propensity score matching on age, sex, education, DM, hypertension, education status, body mass index, fasting glucose, hemoglobin, triglyceride, total cholesterol, eGFR, and uric acid. Adjusted for AST and ALT.

Statistical analysis:

In this study, a propensity analysis was conducted using the nearest neighbor caliper matching without replacement method for each patient. The matching process considered several factors, including age, sex, education, presence of diabetes mellitus (DM), hypertension, body mass index (BMI), fasting glucose levels, hemoglobin levels, triglyceride levels, total cholesterol levels, estimated glomerular filtration rate (eGFR), and uric acid levels.

Table S2. Multivariable logistic regression analysis of subgroup of habits combination associated with hepatitis C virus infection (1:10 matching using propensity score matching)

|  | 1:10matching* |  |  |
| --- | --- | --- | --- |
| Variables | Adjusted OR (95% CI) | *p* |  |
| Alcohol (-) Betel nut (-) Cigarette (-) | Reference |  |  |
| Alcohol (+) Betel nut (-) Cigarette (-) | 1.171 (0.890-1.541) | 0.261 |  |
| Alcohol (-) Betel nut (+) Cigarette (-) | 1.148 (0.589-2.238) | 0.686 |  |
| Alcohol (-) Betel nut (-) Cigarette (+) | 1.027 (0.920-1.147) | 0.631 |  |
| Alcohol (+) Betel nut (+) Cigarette (-) | 1.441 (0.603-3.444) | 0.411 |  |
| Alcohol (+) Betel nut (-) Cigarette (+) | 1.426 (1.191-1.707) | < 0.001 |  |
| Alcohol (-) Betel nut (+) Cigarette (+) | 1.299 (1.066-1.582) | 0.009 |  |
| Alcohol (+) Betel nut (+) Cigarette (+) | 1.697 (1.405-2.050) | <0.0001 |  |

Values expressed as odds ratio (OR) and 95% confidence interval (CI). Abbreviations. HCV, hepatitis C virus.

Adjusted for age, sex, diabetes, hypertension, education status, fasting glucose, hemoglobin, total cholesterol, AST, ALT, eGFR and uric acid. Interactions between the values of alcohol, betel nut, and cigarette were tested using the logistic regression model with an added interaction term (alcohol by betel nut by cigarette) and covariates (*p* for interaction =0.9634).

*1:10 propensity score matching on age, sex, education, DM, hypertension, body mass index, fasting glucose, hemoglobin, triglyceride, total cholesterol, eGFR, and uric acid. Adjusted for AST and ALT. Interactions between the values of alcohol, betel nut, and cigarette were tested using the logistic regression model with an added interaction term (alcohol by betel nut by cigarette) and covariates (*p* for interaction= 0.8280).
